# Bursts of Bipolar Microsecond Pulses Inhibit Tumor Growth

**DOI:** 10.1038/srep14999

**Published:** 2015-10-13

**Authors:** Michael B. Sano, Christopher B. Arena, Katelyn R. Bittleman, Matthew R. DeWitt, Hyung J. Cho, Christopher S. Szot, Dieter Saur, James M. Cissell, John Robertson, Yong W. Lee, Rafael V. Davalos

**Affiliations:** 1 School of Biomedical Engineering and Sciences, Virginia Tech, USA; 2 Department of Radiation Oncology, Division of Radiation Physics, Stanford University, USA; 3 Technische Universität München, Germany; 4 Virginia-Maryland College of Veterinary Medicine, USA.

## Abstract

Irreversible electroporation (IRE) is an emerging focal therapy which is demonstrating utility in the treatment of unresectable tumors where thermal ablation techniques are contraindicated. IRE uses ultra-short duration, high-intensity monopolar pulsed electric fields to permanently disrupt cell membranes within a well-defined volume. Though preliminary clinical results for IRE are promising, implementing IRE can be challenging due to the heterogeneous nature of tumor tissue and the unintended induction of muscle contractions. High-frequency IRE (H-FIRE), a new treatment modality which replaces the monopolar IRE pulses with a burst of bipolar pulses, has the potential to resolve these clinical challenges. We explored the pulse-duration space between 250 ns and 100 μs and determined the lethal electric field intensity for specific H-FIRE protocols using a 3D tumor mimic. Murine tumors were exposed to 120 bursts, each energized for 100 μs, containing individual pulses 1, 2, or 5 μs in duration. Tumor growth was significantly inhibited and all protocols were able to achieve complete regressions. The H-FIRE protocol substantially reduces muscle contractions and the therapy can be delivered without the need for a neuromuscular blockade. This work shows the potential for H-FIRE to be used as a focal therapy and merits its investigation in larger pre-clinical models.

Irreversible electroporation (IRE) is a relatively new focal ablation technique for the treatment of solid tumors^1^. The procedure uses brief high-intensity pulsed electric fields which results in rapid cell death of a targeted volume^2^. Typical treatment protocols involve the insertion of two needle electrodes into the tumor volume. Electrical pulses 50–100 μs in duration, are then delivered in synchrony with the patient’s heartbeat. A total of 80–200 pulses are usually delivered in a typical IRE protocol^3^. The pulsed electrical fields lead to the formation of nano-scale defects in the cell membrane which, above a critical threshold, the cells are unable to recover from. The volume of tumor tissue treated is controlled by adjusting the separation between electrodes, the length of metal exposed on each electrode, and the applied voltage.

IRE is currently being clinically evaluated for the treatment of multiple oncological diseases including pancreatic^4^,^5^,^6^, lung^7^, brain^8^, kidney^9^,^10^,^11^,^12^,^13^, and liver^14^,^15^,^16^,^17^,^18^ cancers. A review positively highlighting the safety and efficacy of these treatments in a clinical setting was recently published by Scheffer *et al.*^19^ IRE appears to be ideally suited for the treatment of tumors less than 3.0 cm with success rates between 93%^15^ and 98%^14^ reported in early clinical studies on hepatic tumors below this size.

IRE may also be effective for treatment of tumors in organs or locations which are sensitive to thermal damage. Complication rates for ablations in pancreatic tissue are significantly lower in IRE (19%) versus radio frequency ablation (28–40%)^19^ due to the non-thermal nature of IRE treatments. Martin *et al.*^4^ recently showed that combinatorial treatment including chemo-, radiation, and IRE therapy improved local progression-free survival times by eight months in patients with primary pancreatic tumors, highlighting the potential of IRE for multimodal tumor therapy. Though limited data is available, both cryo- and radio-frequency ablation techniques appear to have high complication rates in neurological applications due to the sensitive nature of the tissue^20^,^21^. However, recent canine studies by Garcia *et al.*^21^ and Ellis *et al.*^22^ showed the safety of IRE for applications in the brain and its effectiveness against malignant glioma in combination with adjunctive fractionated radiotherapy^22^. These recent advancements highlight the promise of IRE in combinatorial therapy and as a standalone treatment for solid tumors.

One of the key highlights of IRE is the ability to safely treat tumors which are in close proximity to sensitive structures such as large blood vessels^14^, nerve beds^23^, or the ventricles^24^. However, electrically induced muscle contractions are an unintended consequence which may move the electrodes during treatment, resulting in possible complications, and must be considered when treating near these sensitive structures. When improperly managed, organ translocations of 3 to 5 cm have been reported in response to pulse delivery^25^. To prevent this, patients are anesthetized during IRE treatment using a strict protocol which includes the administration of a neuro-muscular blockade (vecuronium or rocuronium) which requires intubation and mechanical ventilation^25^. There is evidence that these neuromuscular blocks interfere with pulmonary, respiratory, and pharyngeal function^26^ and in some cases mild local muscle contraction continues despite these preventative measures^27^.

Minimizing the effects of these muscle contractions has recently become a significant area of research^28^. One promising technique is to optimize the electrode design to limit exposure of nearby muscle tissue to the applied field by minimizing current flow outside of the treatment volume. Golberg and Rubinsky recently demonstrated the use of a current cage, or array of grounding electrodes around a central energized electrode, to minimize the volume of tissue exposed to fields above the muscle contraction threshold^29^. Pulse parameters including shape, polarity, and timing can also be modified to exploit biophysical phenomena which limit muscle contractions. Daskalov *et al.* showed that by delivering eight 50 μs pulses with a 1 ms spacing, patients only experienced a single muscle contraction sensation^30^. The threshold for inducing muscle contractions increases exponentially as pulse duration decreases below 100 μs^31^,^32^ and an alternative approach to mitigating muscle contractions is to deliver short duration pulses on the order of one microsecond.

High-frequency irreversible electroporation (H-FIRE) replaces the single monopolar pulse (Fig. 1A) with a burst of higher frequency bi-polar pules (Fig. 1B). These applied bursts are repeated once per second in synchrony with the heart rate of a clinical patient. Arena *et al.* demonstrated that *in vivo* H-FIRE treatments with 1 or 2 μs pulses eliminated muscle contractions associated with equivalent energy IRE treatments^33^ and bursts of short duration pulses have been theoretically shown to short through epithelial layers and produce more uniform treatment regions in heterogeneous tissues^34^.

The lethal electric field threshold for this H-FIRE protocol has not yet been established and electroporation effects of pulses in the 1 to 100 μs range are still relatively unexplored^35^. Typically, the response of cells in a media suspension has been used as a surrogate for determining the lethal electric field threshold, however, 3D tissue mimics have been found to more accurately represent the thresholds found *in vivo*^36^. These 3D tissue engineered tumor models overcome many of the shortcomings and limitations of cells in suspension through better replication of *in vivo* morphology, and the inclusion of cell-cell and cell-matrix interactions. Additionally, the tissue like nature allows for cells to remain stabilized in the matrix which allows for studies of actual applied electrical field which varies spatially.

This study presents the lethal electric field intensity for a number of H-FIRE protocols as determined in a 3D tissue model. For equivalent energy H-FIRE treatments, we found that the lethal electric field intensity increased from 530 V/cm to 2020 V/cm as the pulse-width was decreased from 50 μs to 250 ns, respectively. We showed that H-FIRE was effective *in vivo* against a murine flank tumor model using bursts containing 1, 2, and 5 μs pulses. In total, 6 of 14 treated mice had no measurable signs of tumors 30 days after treatment and at least one mouse from each protocol reached complete regression. Finally, we show qualitatively that the H-FIRE protocol reduces the extent of muscle contractions in both a murine and equine model, enabling the delivery of the therapy under mild sedation rather than complete anesthetic conditions.

## Materials and Methods

### Collagen Hydrogel Tumor Mimics

PPT8182 murine primary pancreatic tumor cells^37^, shown to replicate human pancreatic cancer in terms of histology, metastasis, and genetic alterations^37^,^38^,^39^,^40^, were used in the 3D tumor platform experiments. Cells were cultured in Dulbecco’s Modified Eagle Medium (DMEM) supplemented with L-glutamine (ATCC, Manassas, VA) containing 10% fetal bovine serum (FBS; Sigma Aldrich, St. Louis, MO) and 1% penicillin/streptomycin (Invitrogen, Carlsbad, CA) at 37 °C in 5% CO_2_ in a humidified atmosphere. All cells were harvested for experiments by trypsinization at 80% confluence.

**Figure 1:**
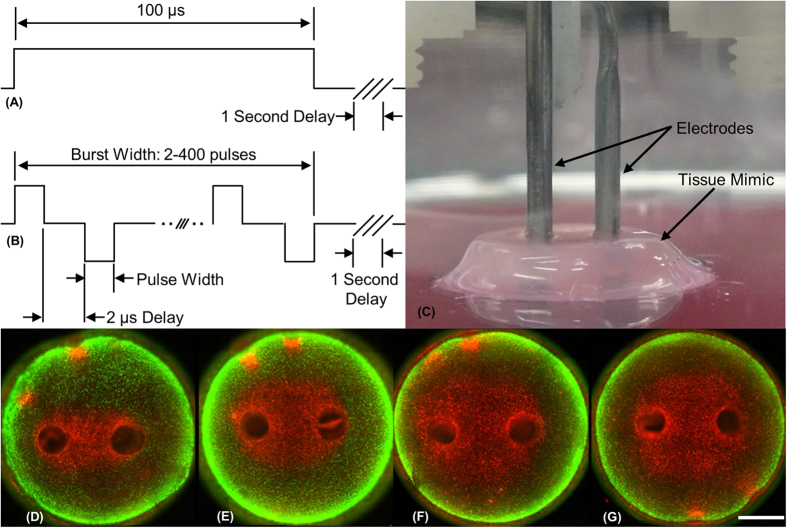
H-FIRE treatment in a 3D tumor mimic. Schematics of (**A**) traditional monopolar IRE pulse and (**B**) high frequency bipolar burst. (**C**) The experimental setup with electrodes inserted into the 3D tissue mimic. Live [green] and dead [red] regions of the tissue mimic after treatment with eighty bursts containing (**D**) 2, (**E**) 24, and (**F**) 50 bipolar 2 μs pulses with a 2 μs delay between alternating pulses. (**G**) Diffuse treatment of 50 bipolar 2 μs pulses with 20 ms between alternating pulses. Scale bar represents 2 mm.

Collagen I hydrogels, shown in Fig. 1C, were produced as described previously^41^. Briefly, Sprague Dawley rat tail tendons were excised and allowed to dissolve under agitation overnight in 10 mM HCl at room temperature. The resulting monomeric collagen suspension was centrifuged at 22,500 × g for 30 min, and the supernatant was decanted and stored at 4 °C until later use. The collagen hydrogels were formed by neutralizing the collagen I in HCl with a buffer containing 10× concentrated DMEM (supplemented with 4.5 g/L glucose, L-glutamine, sodium pyruvate, and sodium bicarbonate; Mediatech Inc., Manassas, VA), 1 N NaOH, and deionized H_2_O to obtain a final concentration of 8 mg/mL at a pH of 7.4. The PPT8182 cells were suspended in the neutralizing buffer at a final seeding density of 1 × 10^6^ cells/mL and then mixed with the collagen I solution. The collagen-cell suspension was pipetted into 10 mm diameter cylindrical molds to achieve a thickness of 3 mm after polymerization. Following a 20 min gelation period at 37 °C, the hydrogels were removed from the molds and cultured in complete media for 18 hours prior to pulse delivery.

### Electronics and Protocols

A custom pulse generation system was used to deliver bursts of bi-polar pulses with constitutive pulse widths of 250 ns, 500 ns, 1 μs, 2 μs, 5 μs, 10 μs, and 50 μs. A 500 Ω resistor was placed in parallel with the load to ensure proper pulse shaping and to protect against delivering pulses to an open circuit. Representative examples of these bursts can be seen in Supplemental Figure 1. Custom electrodes were made from hollow 1.27 mm diameter dispensing needles (Howard Electronic Instruments Inc., El Dorado, KS) with a 2.0 mm edge-to-edge separation distance.

A pilot study was conducted at 540 V_peak_ and a total energized time of 100 μs for all pulse widths. This protocol used 400, 200, 100, 50, 20, 5, or 2 pulses to comprise a burst, with individual pulse durations of 250 ns, 500 ns, 1 μs, 2 μs, 5 μs, 10 μs, or 50 μs, respectively. The ablation zones at 540 V_peak_ for bursts containing pulses 1 μs or less were not well formed ovals surrounding the electrodes. Instead, dead cells occupied small triangular zones which extended, but did not connect between the two electrodes. The electric field intensity changed rapidly in this zone resulting in large variations in the calculation of electric field thresholds. To avoid this, a higher voltage of 650 V was used for the 250 ns, 500 ns, 1 μs and 2 μs groups. To facilitate comparison between groups, a simplified electrical dose formula was used.


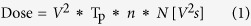


where V is the applied voltage, T_p_ is the pulse width, n is the number of pulses per burst, and N is the number of bursts per treatment which was typically 80. The 540 V_peak_ group had an approximate dose of 2300 V^2^s. At 650 V_peak_, 256, 128, 64, and 32 pulses were used for the 250 ns, 500 ns, 1 μs, and 2 μs groups, respectively. This resulted in an approximate dose of 2200 V^2^s. An additional 2 μs group at 250 V_peak_ with 216 pulses an approximate dose of 2000 V^2^s was also conducted to compare effects of energy and lethal electric field threshold.

To explore the effect of burst energized time, a set of experiments were conducted with 80 bursts containing 2 μs pulses at 540 V. Pulses were repeated 2, 24, or 50 times per burst with a 2 μs inter-pulse delay. To compare ‘diffuse’ and ‘burst’ delivery of pulses an additional group of 50 pulses per second was tested. In this group, one positive and one negative pulse were delivered, with a 2 μs inter-pulse delay, every 20 ms for a total of 80 seconds. This is the only group presented in which a 1 second inter-burst delay was not used.

To explore the effect of treatment time, a set of experiments were conducted with eight bursts. These groups had 2 μs, 50 μs, and 100 μs pulses which were repeated 50, 2, and 1 times per burst, respectively. The experimental parameters are summarized in Table 1. All parameters were repeated a minimum of three (n = 3) times.

### Sample Processing

At 24 hours after treatment, normal culture media was replaced with 2.5 mL of media supplemented with 4 μM Calcein AM (live stain, λ_em_ = 515 nm, Invitrogen, Eugene, OR) and incubated at 37 °C for 30 minutes. Five minutes prior to visualization, the media was supplemented with 75 μL of 1.5 mM propidium iodide (PI; dead stain, λ_em_ = 617 nm, Invitrogen, Eugene, OR) for 5 minutes. Finally, the hydrogels were rinsed with PBS to flush out any unabsorbed dyes and increase the signal to noise ratio. A Leica DMI 6000 fluorescent microscope with a 20x objective (Leica Microsystems Inc., Buffalo Grove, IL) was used to tile a set of images and reconstruct an entire plane of the treated scaffolds just under the surface.

### Analysis of Electric Field Thresholds in Tissue Mimics

Finite element models were created in COMSOL Multiphysics (Version 4.2a, COMSOL Inc., Burlington, MA). The collagen hydrogels were modeled as a 3 mm thick cylinder with a 5 mm radius and conductivity of 1.2 S/m. Cylinders representing the 1.27 mm outer diameter electrodes were offset such that their edge-to-edge distance was equal to 2 mm. Within the solution domain, the Electric Currents module was used to solve for following equations


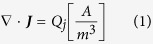



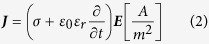



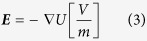


where U is the electric potential, **E** is the electric field, **J** is the current density, Q is the current source, σ is the conductivity, ε_r_ is the relative permittivity, and ε_0_ is the permittivity of free space. The boundaries surrounding one electrode were assigned a constant electrical potential


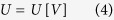


The boundaries of the other electrode were assigned as a relative ground


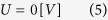


The remaining boundaries were defined as electrical insulation


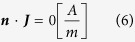


where ***n*** is the normal vector to the surface, **J** is the electrical current.

Changes in temperature due to Joule heating were calculated for 540 V and 100 μs energized time over 80 seconds using a modified duty cycle approach^33^,^42^. The temperature distribution (***T***) was obtained by transiently solving a modified heat conduction equation:


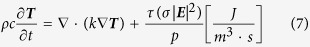


where τ is the pulse duration, *P* is the period of the pulses, *k* is the thermal conductivity, *c* is the specific heat at constant pressure, and *ρ* is the density. Outer boundaries were treated as convective cooling


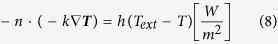


with an exterior temperature (*T*_*ext*_) of 22 °C and a heat transfer coefficient (h) of 25 (W m^−2^ K^−1^). Intermediate time stepping was used to ensure that at least one time step was taken each second. Parameter values for these simulations can be found in Supplemental Table 1. Simulations at 540 V showed that that thermal effects resulted in a negligible impact on the electric field distribution and changes in conductivity due to temperature increases were neglected in subsequent models to minimize computational time. Changes in conductivity due to electroporation were similarly neglected due to the low concentration of cells within the scaffold. To replicate the values measured experimentally, the voltage on one electrode was swept between 470–700 V, in steps of 10 V, and the other was held at ground.

Tiled images near the surface of the hydrogels (representative examples in Fig. 1D,E) were examined using ImageJ (version 1.43u, National Institutes of Health, USA). The width and height of the region of cells that had taken up PI (dead region) was measured. These values were then correlated to the electric field intensity from the numerical simulations to determine the electric field threshold required for cell death^36^. Statistical analysis of the data was completed using JMP (Version 10.0 Pro, SAS Institute Inc., Cary, NC) with a confidence level of 99% (α = 0.01) unless otherwise noted.

### Murine tumor model

This experimental protocol was approved by the Virginia Tech Institutional Animal Care and Use Committee. All methods were carried out in accordance to the approved institutional guidelines. 6–7 week old Hsd:Athymic Nude-Foxn1^nu^ male mice (Harlan, Dublin, VA were inoculated subcutaneously in the dorsolateral flank region with human glioblastoma cells (DBTRG-05MG) while anesthetized by inhalation of 3% isoflurane (Abbott Laboratories, Abbott Park, IL). Mice were housed in individually ventilated cages in groups of five under specific pathogen free conditions and allowed access to sterilized water and food *ad libitum*. Prior to inoculation, cells were cultivated using standard techniques in DMEM (High-glucose supplemented with L-glutamine; Thermo Scientific, Logan, UT) containing 10% FBS and 1% penicillin/streptomycin. Upon reaching 80% confluence, cells were suspended at a concentration of 5 × 10^6^ cells/mL in an 85/15 mixture of PBS and Matrigel (BD Biosciences, San Jose, CA). 200 μL aliquots of this final suspension was used for each injection (1 × 10^6^ cells total).

Tumor growth was measured over time using calipers, and volumes (v) were calculated according to the modified ellipsoid formula^43^:


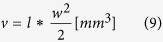


where *l* is the length of the longitudinal diameter and *w* is the width of the transverse diameter. Tumors were treated when the greatest diameter reached approximately 5 mm. Mice were anesthetized following the same isoflurane inhalation protocol, and the skin over the tumor was prepped with 70% isopropyl alcohol. Then, custom steel needle electrodes (0.4 mm Ø) were advanced into the center of the tumor. A 0.4 cm spacing (center-to-center) was used in all treatments. In all HFIRE treatment groups, the pulse generation system was set to deliver its maximum 1000 V_peak_ output. The energized time per burst was fixed to 100 μs and bursts were delivered with a repetition rate of 1 Hz for 2 minutes. Mice were assigned to treatments with constituent pulse widths of 5 μs (n = 8), 2 μs (n = 2), 1 μs (n = 4) or sham control (n = 4) with specific pulse parameters shown in Table 2. Each treatment was video recorded to qualitatively evaluate the extent of muscle contractions. To compare these results with clinically implemented IRE protocols, an additional mouse received 100 μs mono-polar pulses at 200 V.

Following treatment, topical antibiotic ointment was applied to the needle insertion wounds. Mice were removed from anesthesia and provided 5 mg/kg ketoprofen analgesic diluted in 1 mL sterile saline solution for recovery. The mice were euthanized 30 days post-treatment or earlier for humane reasons if the tumor volume reached 800 mm^3^. Statistical significance between groups was determined at day 30 using a one sided Student’s T-test with unequal variances and alpha = 0.1.

Samples of any present tumor tissue were excised and sectioned for processing. Representative tissues were preserved in 10% neutral buffered formalin and embedded in paraffin. Formalin preserved paraffin embedded samples were sectioned and processed for histology using hematoxylin and eosin (H&E) staining. All photomicrographs were obtained with a Leica DMI 6000 inverted microscope.

## Results

### H-FIRE pulse width, pulse number, and total energized time affect the lethal electric field threshold

**Figure 2:**
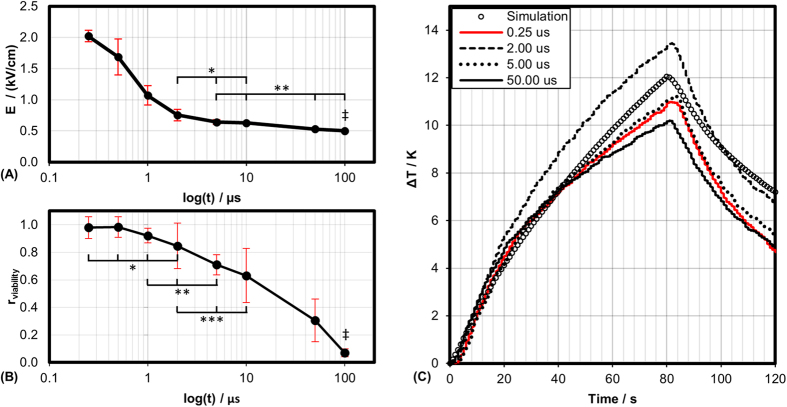
Non-thermal lethal thresholds are dependent on pulse-width. (**A**) Lethal electric field threshold for PPT cells in tissue mimic for 2200 V^2^s dose. (**B**) Relative viability of PPT cells in media suspension after treatment with 1500 V/cm; Data in (**B**) from Sano *et al.*^47^ and ‡ (**A,B**) from Arena *et al.*^37^. (**C**) Temperature profile at center of tissue mimic as measured experimentally and predicted numerically. (*, α = 0.01), (**, α = 0.05), and (***, α = 0.1).

Typical IRE treatments involve the delivery of 80 monopolar pulses, each 100 μs in duration at a repetition rate of 1 Hz. Using the PPT8182 cell line and the same tissue mimic, Arena *et al.* found that the lethal threshold for this standard protocol is 501 V/cm^36^. Figure 2A shows the lethal threshold when the monopolar pulse is replaced by a burst of bipolar pulses with an equivalent electrical dose. The lethal electric field thresholds were found to be 2022, 1687, 1070, 755, 640, 629, and 531 V/cm for bursts containing 0.25, 0.5, 1, 2, 5, 10 and 50 μs pulses, respectively.

The temperature profiles measured were well correlated to those predicted numerically (Fig. 2C). Simulations of these pulses predict a temperature increase of approximately 12 °C at the center of the tissue mimic after 80 pulses were delivered. Experimentally, the average temperature increase across all groups was 14.4 ± 2.2 °C. Experiments were conducted at room temperature and the maximum temperature measured experimentally was 34.8 °C. The largest variation in maximum temperature, 3.2 °C, occurred between the 2 μs and 50 μs groups.

**Figure 3:**
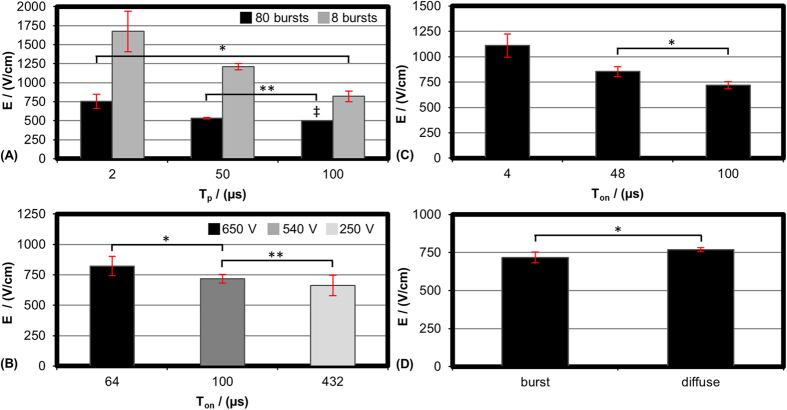
Exploration of treatment parameters. Lethal electric field threshold for (**A**) 540 V and 100 us energized time per burst with 8 or 80 bursts per treatment. 2 and 50 μs groups contained bipolar pulses, 100 μs group had monopolar pulses (**B**) 2 μs group at 250, 540, and 650 V with equivalent energy per burst (**C**) 2 μs group at 540 V with 4, 48, or 100 μs energized per burst (**D**) 2 μs group at 540 V where inter-burst delay was 1 s [burst] or 20 ms [diffuse]. (**B–D**) Treatment groups received 80 bursts or treatment for 80 seconds [diffuse group]. ‡ Data from Arena *et al.*^37^. (*, α = 0.01) and (**, α = 0.05).

Treatments with 8 and 80 bursts were conducted for bursts with 2 and 50 μs pulses. For comparison, treatments with either 8 or 80 monopolar pulses 100 μs in duration were conducted (Fig. 3A). The thresholds for 8 pulses were found to be 1675, 1211, and 820 V/cm, for the 2, 50, and 100 μs groups, respectively. The corresponding thresholds for 80 pulses were found to be 756, 531, and 501 V/cm.

To explore the limitations of our equivalent dose approximation, eighty bursts held constant with 2 μs pulses were delivered at three different voltages: 250, 540, and 650 V. For these cases, each burst contained 216, 50, and 32 pulses, resulting in approximate doses of 2000, 2300, and 2200 V^2^s, respectively. The threshold for cell death for these treatments were 663, 718, and 822 V/cm (Fig. 3B). The 250 and 650 V groups were found to be statistically different with a 99% confidence level (α = 0.01).

For bursts with 2 μs pulses, when the voltage was held constant at 540 V, but the energized time per burst was decreased from 100 to 48 or 4 μs, the electric field threshold was found to increase from 718 V/cm to 855 and 1110 V/cm, respectively (Fig. 3C). The difference between 100 and 48 μs was not statistically significant.

Figure 3D shows the effect of inter-pulse delay on lethal electric field threshold. At 540 V, the inter-pulse delay between 2 μs pulses was increased from 2 μs to 200 μs. Similar to the ‘burst’, this ‘diffuse’ treatment was energized for 100 μs per second and this waveform was delivered for 80 seconds. This change in inter-pulse delay resulted in an increase in electric field threshold from 718 V/cm to 770 V/cm; this difference was not statistically different.

### H-FIRE treatment inhibits tumor growth *in vivo*

**Figure 4:**
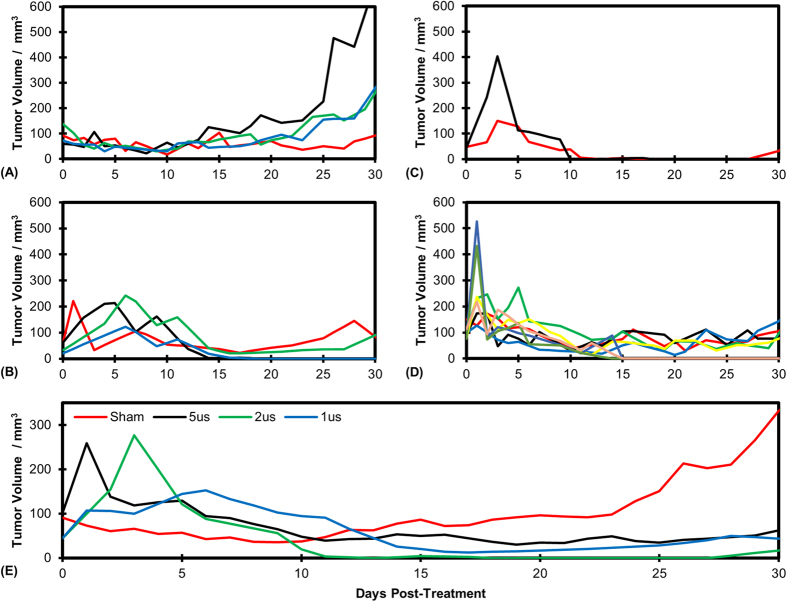
H-FIRE treatment inhibits tumor growth. Tumor volume as a function of days post treatment for (**A)** Sham group, (**B**) 1 μs group (**C**) 2 μs group, and (**D**) 5 μs group. (**E**) Volume of tumors averaged across all mice for each treatment group.

At the time of treatment, tumors were on average 91, 101, 45, and 44 mm^3^ for the sham, 5 μs, 2 μs, and 1 μs groups. Thirty days post-treatment, these averages had changed to 332, 62, 16, and 44 mm^3^ (Fig. 4). Three of the four sham tumors more than doubled in size by day 30 (Fig. 4). The fourth did not significantly increase in size and measured 92 mm^3^ at the conclusion of the study. Tumors in the 1, 2, and 5 μs group (Fig. 4B–D) exhibited varying increases in size over days 1–5 before regression was observed. The 1 μs group had two complete regressions at the end of the study. The other two tumors measured 85 and 91 mm^3^ on day 30. The 2 μs group had 1 complete regression and the other tumor measured 32.9 mm^3^ on day 30 (Fig. 4C). The 5 μs group had 3 complete regressions. The remaining tumors had volumes of 77, 77, 97, 106, and 144 mm^3^. Figure 4E shows the average tumor volumes for each treatment group over the 30 day trial. All treatment groups achieved a statistically significant reduction in tumor volume versus control on day 30.

**Figure 5:**
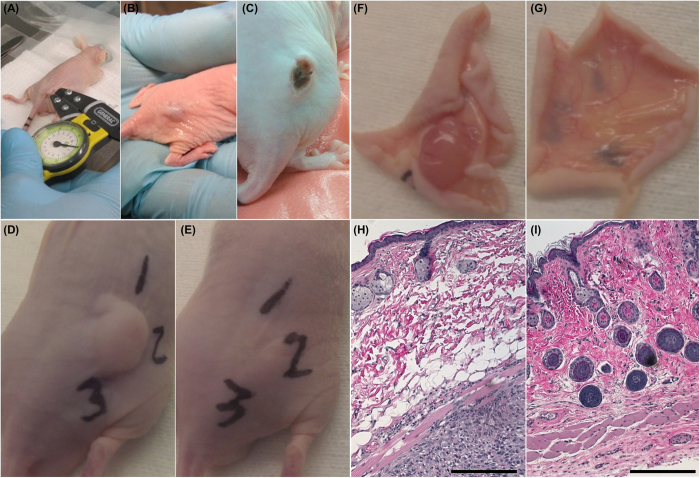
*In vivo* H-FIRE treatments. (**A**) Pulses were delivered through needles inserted into the tumor. (**B**) Immediate tumor whitening and (**C**) scab formation after 24 hours were observed after most treatments. Representative end point images from (**D,F**) the sham group and (**E,G**) the 5 μs group show the existence and absence of subcutaneous tumor 30 days post-treatment, respectively. Numbers written on the surface of the skin are for tissue orientation during histological preparation. (**H**) Sham mouse superficial skin (top of image) and underlying tumor (bottom of image). (**I**) 5 μs treated mouse superficial skin (top of image) and underlying musculature (bottom of image). Scale bars represent 250 μm.

Immediately following *in vivo* treatment, whitening of the tumor occurred. This is associated with reduced blood flow and the beginning stages of edema (Fig. 5B). This characteristic anti-vascular effect of electroporation-based therapies has been utilized in electro-chemotherapy (ECT) to treat bleeding metastasis^44^. Due to the use of uninsulated electrodes, the skin overlying the tumor was killed in conjunction with the tumor. This resulted in scab formation (Fig. 5C) within 1 day post-treatment which typically resolved within two weeks. Endpoint images taken immediately prior to and following tissue harvesting (Fig. 5D–G) show evidence of complete tumor regression 30 days after H-FIRE treatment.

Figure 5H,I shows histological sections from the study endpoint of a mouse in the sham group (Fig. 5H) and 5 μs treatment group (Fig. 5I). Despite the fact that no measurable tumor was observed in the treated mouse, pockets of viable glioblastoma cells were present surrounding blood vessels located above the musculature. Similar features were seen in the sham mouse, with the addition of a viable tumor mass beneath the muscle layer. Cells comprising the viable tumor display a large nucleus surrounded by a well-marked cytoplasm and well-defined cell membrane. Additionally, there is evidence of healthy vasculature along the margin of the tumor at the interface between the muscle and fat layer.

### The H-FIRE Protocol Reduces Muscle Contractions *In Vivo*

During the murine *in vivo* experiments, some muscle contractions were observed in all treatment groups. Supplemental video 1 compares 5 μs bursts with 100 μs mono-polar pulses at 1000 V and 200 V, respectively. Qualitatively, muscle contractions occurred to a lesser extent in treatments with bi-polar bursts of pulses between 1 and 5 μs than occurred in treatments with mono-polar 100 μs pulses. Delivery of 200 V, 100 μs mono-polar pulses resulted in significant muscle contractions of both hind limbs. Less intense muscle contractions were observed in the bi-polar treatments, typically confined to the proximal limb, which could be further minimized by gently lifting the electrodes to pull the tumor away from nearby muscle tissue.

Mice represent the smallest possible animal model and their bodies have relatively little inertia, resulting in some movement despite the use of bi-polar bursts. Supplemental video 2 shows a comparison of 100 μs mono-polar pulses and bursts of 5 μs pulses in the treatment of spontaneous tumors in an equine model. At 400 V, the 100 μs pulses induce such strong muscle contractions that complete anesthesia is necessary carry out the procedure, while the animal is in a prone position. In contrast, 1000 V treatments with bursts of 5 μs pulses are well tolerated with light sedation and local anesthesia while the patient is in an orthostatic position.

## Discussion

For bursts of bipolar pulses, the electric field threshold required to induce cell death is inversely correlated to the duration of the constitutive pulses (Fig. 2A). The lethal threshold increases slightly as pulse duration is decreased from 50 μs to 2 μs. The threshold for cell death for bursts with 1 μs pulses is approximately double the threshold for bursts with 50 μs pulses and 250 ns pulses have a threshold approximately four times greater than the 50 μs treatments. The treatments shown in Fig. 2A all received equivalent doses in 80 bursts.

Figure 2B shows data adapted from Sano *et al.*^45^ and Arena *et al.*^36^ for PPT8182 cells suspended in media and exposed to 80 monopolar 100 μs pulses or 80 bi-polar bursts with pulses between 250 ns and 50 μs (100 μs energized per burst) with a 1500 V/cm voltage-to-distance ratio. In suspension, bursts with 2 μs or shorter pulses do not affect cell viability. In contrast, 1500 V/cm is sufficient to kill all of the cells in the tissue mimics for bursts with pulses 1 μs or longer.

When the cells are in suspension, they take on a more spherical appearance. In contrast, when grown in the 3D tissue mimics they begin to stretch out and obtain a more natural phenotype. *In vivo*, IRE is typically observed in regions which are exposed to approximately 500–750 V/cm 24,46,47 and the field strengths predicted in these 3D tissue mimics are more likely to represent the *in vivo* thresholds for bipolar bursts. However, extensive *in vivo* evaluation is still needed to determine how these thresholds compare to those necessary to ablate complex heterogeneous tissues such as pancreatic tumors which contain healthy and malignant cells, vasculature, ductile systems, and connective tissue.

Electrogenetransfer (EGT) and ECT protocols typically employ 8 pulses with the goal of permeabilizing the cell membrane, but not inducing cell death. Figure 3A shows that there is a significant difference between 8 monopolar 100 μs pulses and bipolar 50 μs bursts. This is interesting because these groups were not significantly different when the burst number was increased to 80. Increasing the number of pulses reduced the lethal electric field threshold significantly for all groups. Between 8 and 80 pulses, the thresholds drop by 920 V/cm (55%), 679 V/cm (56%), and 319 V/cm (39%) for the 2 μs bipolar, 50 μs bipolar, and 100 μs monopolar groups, respectively. Interestingly, the lethal thresholds for 80 bursts with 2 μs pulses was the same as 8 monopolar 100 μs pulses. Though not investigated here, the use of bi-polar pulses may allow investigators to treat larger volumes using EGT or ECT without deleterious lethal effects.

Protocols with 1 μs, 500 ns, and 250 ns failed to produce connected lesions in the tissue mimics when the voltage was set to 540 V and the energized time per burst was 100 μs. This made it difficult to accurately calculate the lethal electric field threshold. In our initial pilot study, we found that increasing the voltage to 650 V while delivering 80 pulses with 100 μs energized time resulted in thermal denaturing of the collagen matrix. Arena *et al.* associated collagen denaturation during IRE with temperatures greater than 45 °C^36^. Reducing the energized time to 64 μs at 650 V, a similar dose to 540 V and 100 μs, resulted in well-formed oval shaped lesions for all groups. We used this higher voltage, equivalent dose protocol for all groups with 1 μs pulses and shorter.

In Fig. 3B we investigated the validity of this equivalent dose hypothesis using bursts with 2 μs pulses, which formed connected lesions at the lowest voltage tested, 250 V. There is no statistical difference between equivalent dose protocols at 650 V and 540 V nor between 540 V and 250 V protocols with a 99% confidence level (α = 0.01) and there is no statistical difference between the three groups with a 95% confidence level (α = 0.05). This indicates that in the 3D tumor mimic model, an equivalent dose approximation is sufficient for comparing protocols.

It is unclear how far outside this range (250–650 V) the equivalent dose hypothesis is valid. However, clinical IRE systems are currently limited to outputs of 2700 V. At this voltage, a burst energized for 4 μs would have an equivalent dose and a lethal threshold of approximately 750 V/cm (the average of values from Fig. 3B). Figure 3C shows that when bursts are energized for 100 μs versus 4 μs, there is 35% reduction in the lethal threshold. If these two effects are additive, we can hypothesize that a protocol with 80 burst of 2 μs pulses, energized for 100 μs per burst (Dose ≈ 58,000 V^2^s), will have a lethal threshold of approximately 460 V/cm. This indicates that H-FIRE treatments should be capable of creating similar ablation volumes as the clinical systems currently employed. However, extensive *in vivo* testing and measurement of ablation volumes will be required to validate this.

Previous *in vivo* IRE experiments on murine tumor models required the application of pulses with 1000 *V*_*peak*_ amplitude or greater to obtain complete regression of similar sized tumors. Neal *et al.*^48^ achieved complete regression in 5 of 7 mice when 100 monopolar pulses, each 100 μs in duration and 1300 *V*_*peak*_ (5600 V/cm) were applied through a bi-polar probe with a 2.3 mm electrode spacing. Al-Sakere *et al.*^49^ achieved complete regression in 12 of 13 mice when 80 pulses, each 100 μs in duration and 1000 *V*_*peak*_ (2500 V/cm) were applied between plate electrodes spaced 4 mm apart.

To mimic the clinical protocol, treatments in this study were applied through two needle electrodes. A spacing of 0.4 cm was used to maximize coverage of the tumors while accounting for the 1000 *V*_*peak*_ limit of our pulse generation system. The 0.4 mm diameter electrodes used in these *in vivo* experiments were significantly smaller than the 1 mm diameter electrodes used clinically and the 1.27 mm electrodes used in the tumor mimics. Electrode diameter is closely linked to the electric field distribution and smaller electrodes will produce a smaller ablation zone. To account for this, the number of bursts delivered was increased to 120 to provide the best possible outcomes while avoiding extensive thermal heating effects. Gross and histological examination did not indicate any scar formation from thermal damage.

In the treated groups, the measured tumor volume increased over the first 1–5 days post treatment. The formation of a scab along with the occurrence of edema may have led to an overestimation of tumor volumes during short-term follow-up. Within two weeks after treatment delivery, scabs resolved and evidence of tumor regression was observable.

This treatment protocol inhibited tumor growth. The average tumor volumes in the treatment groups were statistically significantly smaller than control at the end of the study. Due to the limited time-span of the IACUC protocol, it is unclear if the tumors would have entered an exponential growth period post-treatment and we were unable to obtain Kaplan-Meier survival curves. In total, 6 of 14 treated mice had no measurable signs of tumors 30 days after treatment and all protocols were able to achieve some complete regressions. Future work should include a long-term study to monitor tumor regression over the lifetime of the animals.

Histological examination of some treated animals revealed pockets of neoplastic cells superficial to the muscle fascia in the dermal layers. This is indicative of under-treatment and it is possible that better regression results can be obtained by using a protocol with a higher applied voltage, increased number of bursts, or higher energized time per burst. It should be noted that the work presented by Al-Sakere did not obtain a 100% regression rates, however, their protocol has been successfully adapted to human clinical applications with promising results. Neal *et al.* observed improved progression free survival times for immunocompetent mice, compared to immunocompromised mice, when tumors were treated with 200 × 100μs mono-polar pulses^50^. CD3+ immune cells were observed to infiltrate the regions between live and dead tumor cells. Additionally, immunocompetent mice re-challenged with tumors 18 days after their initial treatment displayed significantly reduced cell growth in the second tumor. Future work will be necessary to examine if this systemic immune response following IRE protocols is present following H-FIRE protocols.

Qualitatively, bursts of 1–5 μs pulses significantly decreased the muscle contractions observed in murine and equine models of disease. We previously demonstrated quantitatively that the transition from long duration mono-polar pulses to bursts of bipolar pulses eliminates muscle contractions during the ablation of healthy rodent brain tissue even when electric field intensities of 2000 V/cm are employed^33^,^51^. In contrast, mono-polar 100 μs pulses induced measureable muscle contractions at 500 V/cm^33^. Rogers *et al.* showed that the threshold for muscle contractions, of gastrocnemius muscles, increased from 1.83 V/cm to 112 V/cm when pulse duration was decreased from 100 μs to 1 μs^32^, a 61x increase. This indicates that shorter pulses are much less efficient at inducing muscle contractions. In contrast, we show here that the lethal threshold for bursts of 1 μs pulses is only 2.1× higher than for mono-polar 100 μs pulses. The significant increase in muscle contraction threshold paired with a relatively small increase in lethal threshold indicates that clinically relevant ablations can be created without inducing the extreme muscle contractions seen in typical IRE procedures, possibly eliminating the need for anesthetic paralytics.

Golberg *et al.* recently demonstrated that, for IRE pulses, large blood vessels distort the local electric field and protect cells in the region, resulting in pockets of viable cells surrounding the vessel^52^. Arena *et al.* showed numerically that bursts of shorter pulses (0.5–2 μs) pulses are capable of penetrating epithelial layers and producing more uniform electric fields in heterogeneous tissues^34^. Additionally, Bhonsle *et al.* experimentally showed that the electric field distribution during H-FIRE pulses more closely matches the analytical solution than traditional IRE pulses^53^. These combined results indicate that H-FIRE pulses may be less susceptible to distortions due to large vessels in the treatment field, however, experimental validation of this hypothesis is necessary. The results of this *in vivo* pilot study warrant further exploration of H-FIRE as a complementary clinical tool. Large animal studies using clinical electrodes and a higher voltage pulse generator should be conducted to determine the maximum ablation sizes achievable using H-FIRE. Additional equivalent energy studies in pancreatic tissue may help illuminate the extent to which H-FIRE pulses can short through complex heterogeneous tissues.

## Conclusion

This study shows the differences in lethal threshold for IRE and H-FIRE protocols. Despite delivering equivalent doses, bursts with shorter constituent pulses require higher electric field strengths for ablation. The number of bursts, energized time per burst, and pulse duration are all significant factors affecting the lethal threshold. Using 80 bursts we found that 1, 2, and 5 μs pulses had electric field thresholds of 1070, 755, and 640 V/cm. When 120 bursts were delivered *in vivo*, these pulses had similar effects on tumor volume. All mice treated with H-FIRE tolerated the therapy well and experienced a significant reduction in tumor volume when compared to untreated controls. Each group attained at least one complete regression. The extent of muscle contractions during H-FIRE treatment was observably less than IRE treatments and safety studies in equine models demonstrate that these protocols can be administered under mild sedation conditions. This study provides strong evidence that H-FIRE can be used for tumor ablation and future investigation is warranted.

## Additional Information

**How to cite this article**: Sano, M. B. *et al.* Bursts of Bipolar Microsecond Pulses Inhibit Tumor Growth. *Sci. Rep.* **5**, 14999; doi: 10.1038/srep14999 (2015).

## Supplementary Material

Supplementary Information

Supplemental Video 2

Supplemental Video 1

## Figures and Tables

**Table 1:** Tissue mimic experimental parameters (*data from Arena *et al.* 37).

Pulse Width [μs]	Voltage [V]	Pulses per Burst	Delay [μs]	On-Time per Burst [μs]	Bursts	Dose [V^2^·s]
0.25	650	256	2	64	80	2163.2
0.5	650	128	2	64	80	2163.2
1	650	64	2	64	80	2163.2
2	650	32	2	64	80	2163.2
2	540	50	2	100	80	2332.8
5	540	20	2	100	80	2332.8
10	540	10	2	100	80	2332.8
50	540	2	2	100	80	2332.8
100*	540	1	—	100	80	2332.8
2	540	50	2	100	8	233.3
2	650	32	2	64	8	209.7
2	540	2	2	4	80	93.3
2	540	24	2	48	80	1119.7
2	540	50	200	100	80	2332.8
2	250	216	2	432	80	2021.1
50	540	2	2	100	8	233.3
100	540	1	—	100	8	233.3

**Table 2:** Treatment matrix for mouse tumor ablation.

Group	Pulse Width (μs)	Pulses per Burst	Bursts	Voltage (V)	Dose (V^2^/s)
1 (*n* = 8)	5	20	120	1000	12000
2 (n = 2)	2	50	120	1000	12000
3 (n = 4)	1	100	120	1000	12000
2 (*n* = 4)	Sham	—	—	—	—
