# Supplementary material for: Bursts of Bipolar Microsecond Pulses Inhibit Tumor Growth
Source: Sci Rep. 2015 Oct 13;5:14999. doi: 10.1038/srep14999 (PMC4602310; doi:10.1038/srep14999)
Supplement: Supplementary Information [file srep14999-s1.pdf]

Bursts of Bipolar Microsecond Pulses Inhibit Tumor Growth

Michael B. Sano[1,2,*], Christopher B. Arena[1], Katelyn Bittleman[1], Matthew R. DeWitt[1], Hyung J. Cho[1], Christopher C. Szot[1], Dieter Saur[3], James M. Cissell[4], John Robertson[1],Yong W. Lee[1], Rafael V. Davalos[1]

1. School of Biomedical Engineering and Sciences, Virginia Tech
2. Department of Radiation Oncology, Division of Radiation Physics, Stanford University
3. Technische Universität München
4. Virginia-Maryland College of Veterinary Medicine

Materials and Methods

Evaluation of Muscle Contractions in a Large Animal Model

This experimental protocol was approved by the Virginia Tech Institutional Animal Care and Use Committee. All methods were carried out in accordance to the approved institutional guidelines. Experiments to assess the safety of the HFIRE protocol and the extent of muscle contractions were conducted in an equine model of spontaneous disease. This study was approved by the Virginia Tech Institutional Animal Care and Use Committee. Patients presented with tumors which were histologically assessed as squamous cell carcinoma, equine sarcoid, or equine malignant melanoma. Patients receiving the HFIRE protocol were mildly sedated with a combination of detomidine (10-30 µg/kg) and butorphanol (0.1 mg/kg) to facilitate examination and treatment while standing . Two 1.2 mm diameter electrodes (AngioDynamics, Latham, NY) with a 0.5 cm exposed metal tip, were inserted into the tumors with a 0.5 cm center-to-center spacing. The output of the pulse generation system was set to 1000 V, its maximum output. The burst protocol consisted of 20x alternating 5 µs pulses. Burst output was synchronized with the patient's heartbeat, approximately 30-40 BPM, and were delivered for three minutes.

To qualitatively compare the HFIRE protocol with the clinical IRE protocol, an additional patient received one hundred 100 µs pulses with an amplitude of 400 V. Muscle contractions due to IRE prevented the use of mild sedation on standing patients. This patient was instead placed under

complete anesthesia and treated in a prone position. Each treatment was video recorded to qualitatively evaluate the extent the extent of muscle contractions.

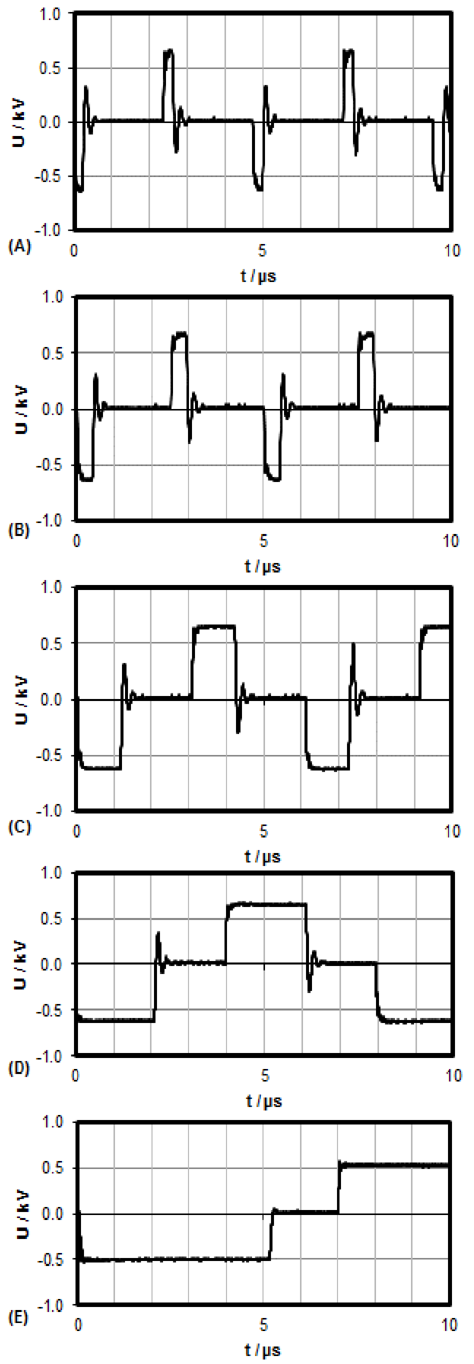

Supplemental Figure 1: Representative examples of the bipolar pulse bursts used experimentally. Bursts contain pulses (A) 250 ns, (B) 500 ns, (C) 1 µs, (D) 2 µs, and (E) 5 µs in duration. A 2 µs delay is implemented between changes in polarity.

Supplemental Table 1: Parameters used in finite element models

Parameter	Tissue Mimic	Well Plate	Electrodes	Unit
σ	1.2	1 x 10^{-16}	2.22 x 10^{6}	$S\ /\ m$
k	0.6	0.14	14	$W\ /\ (m \cdot K)$
ρ	997.8	1050	7900	kg/m^3
c_p	4181.8	1300	477	$J/(kg \cdot K)$

Figure Legends

Supplemental Figure 1: Representative examples of the bipolar pulse bursts used experimentally. Bursts contain pulses (A) 250 ns, (B) 500 ns, (C) 1 μs, (D) 2 μs, and (E) 5 μs in duration. A 2 μs delay is implemented between changes in polarity.

Supplemental Video 1: Representative muscle contractions induced by H-FIRE and IRE pulses. [Left] A flank tumor treated with 1000 V bi-polar bursts of 5 μs pulses. Muscle contractions are confined to the proximal limb and can be further mitigated by lifting the tumor away from the adjacent muscle tissue. [Right] A flank tumor treated with 200 V mono-polar 100 μs pulses. Muscle contractions occur in in the proximal and distal hind limbs.

Supplemental Video 2: H-FIRE pulses enable treatment with mild sedation and local anesthesia. Equine patients with spontaneous tumors on their jaws were treated with either a 1000 V H-FIRE protocol or a 400 V IRE protocol. [Left] A patient under mild sedation with local anesthetic is treated with a series of 1000 V bi-polar bursts of 5 μs pulses. The patient may have experienced some sensation as indicated by swallowing in response to some pulses. However, the treatment was well tolerated and the patient was distracted by gently scratching its face. [Right] A patient receiving 400 V mono-polar 100 μs pulses. Intense muscle contractions induced by this protocol required the patient to be treated while fully sedated.
